# Biocontrol of Carp: The Australian Plan Does Not Stand Up to a Rational Analysis of Safety and Efficacy

**DOI:** 10.3389/fmicb.2019.00882

**Published:** 2019-04-30

**Authors:** Maxime Boutier, Owen Donohoe, R. Keller Kopf, Paul Humphries, Joy A. Becker, Jonathan Marshall, Alain Vanderplasschen

**Affiliations:** ^1^Department of Parasitic and Infectious Diseases, University of Liège, Liège, Belgium; ^2^Bioscience Research Institute, Athlone Institute of Technology, Athlone, Ireland; ^3^School of Environmental Sciences, Institute for Land Water & Society, Charles Sturt University, Albury, NSW, Australia; ^4^Faculty of Science, School of Life and Environmental Sciences, The University of Sydney, Camden, NSW, Australia; ^5^Queensland Department of Environment and Science, Water Planning Ecology, Brisbane, QLD, Australia; ^6^Australian Rivers Institute, Griffith University, Nathan, QLD, Australia

**Keywords:** biological control, Cyprinid herpesvirus 3 (CyHV-3), safety, efficacy, risks, invasive species, ecosystem restoration

## The Australian Plan for Biocontrol of Invasive Carp Using Cyprinid Herpesvirus 3

Common carp (*Cyprinus carpio*), hereafter referred as carp, is one of the most widely cultured fish produced for food globally. It was introduced into Australia for aquaculture in the mid-nineteenth century (Lintermans, 2004). Following floods in the 1970's, carp became abundant and widespread in many Australian waterways where it has come to comprise a large proportion of the fish fauna of these ecosystems (Koehn, 2004). To reduce carp population abundance and aid native species recovery, the Australian Government is evaluating the release of cyprinid herpesvirus 3 [CyHV-3, also known as koi herpesvirus (KHV)] presumed to be absent from Australia (NCCP, 2017). CyHV-3 emerged in the mid 1990's and since that time has devastated carp production worldwide (Boutier et al., 2015). Due to its economic impact on aquaculture, the Word Organization for Animal Health requires notification when the virus is identified.

In a recent opinion letter published in Frontiers in Microbiology, McColl et al. (2018) presented a series of arguments in favor of the use of CyHV-3 as a biocontrol agent against invasive carp in Australia. Here, we summarize key scientific evidence that raises concerns about the safety and efficacy of CyHV-3 as a biocontrol agent. Our review is specifically oriented to clarify misconceptions and omissions by McColl et al. (2018). A multidisciplinary review of the proposed biocontrol plan in achieving the aims of carp control in Australia was summarized recently (Kopf et al., 2019). It suggests that a more detailed cost-benefit analysis is essential before urging the release of CyHV-3 (Kopf et al., 2019).

## Potential Safety Concerns Associated With the Use of CyHV-3 as a Biocontrol Agent

Following carp mortality caused by CyHV-3, decomposing dead fish could itself lead to undesirable environmental damage (Lighten and van Oosterhout, 2017). A detailed analysis of this indirect risk vs the potential benefits of the use of CyHV-3 is published elsewhere (Kopf et al., 2019). The virus may also cause direct deleterious effects by infection of non-target species.

Herpesviruses have mainly evolved by co-speciation with their host, leading to a narrow host range restricted to their natural host species and closely related species (Waltzek et al., 2009). Consistently, it has been demonstrated that CyHV-3 causes mortality only in common and koi carp. However, the ability of the virus to establish subclinical infection in non-natural host species, to persist and to be transmitted from the latter to naïve cohabitant carp has been suggested based on PCR analyses (Kempter et al., 2012; Fabian et al., 2013, 2016). The biological relevance of these data should be taken with caution as the signals observed were weak and the transmission to sentinel carp was never associated with the expression of CyHV-3 disease. The risk of CyHV-3 infecting non-target species of Australia was investigated in limited experimental conditions in a recent paper (McColl et al., 2017). Importantly, the results of that study indicate that CyHV-3 may have induced mortality in at least some native fish species [e.g. silver perch (*Bidyanus bidyanus*)] in Australia. McColl et al. (2017) ignored the potential role of CyHV-3 in the deaths observed (including in the groups where mortality was significantly higher in infected than in mock-infected groups) by presenting data suggesting that viral transcripts could not be detected in dead fish. The possibility of technical issues or the hypothesis that CyHV-3 could induce death in non-natural host species through a non-replicative pathogenesis, as observed for other herpesviruses (e.g., alcelaphine herpesvirus 1) (Palmeira et al., 2013), were not addressed. For these reasons, the data published by McColl et al. (2017) cannot be used to conclusively support the safety of CyHV-3 as a biocontrol agent with regards to effects on non-target species in Australian waterways. Further experiments are still required to investigate this question.

While phylogenetic analyses demonstrate that herpesviruses have mainly evolved by co-speciation with their host, they also suggest that host-jumps have occurred on several occasions throughout evolution (Waltzek et al., 2009). The proposed biocontrol plan consisting of a continental-scale release of a fully virulent strain may represent an unprecedented scenario favoring the selection of a mutant able to infect non-natural host species.

## Factors Likely to Negatively Affect the Efficacy of CyHV-3 as a Biocontrol Agent

### Natural Resistance and Fecundity of Carp

The existence of genetic polymorphisms conferring resistance to a biocontrol agent associated with the high fecundity of the target species represent major obstacles to long-term efficacy of that biocontrol measure. While preliminary experiments performed in laboratory conditions demonstrated that at least some of the wild Australian carp are susceptible to CyHV-3 disease (McColl et al., 2017), the same study also demonstrated the existence of rather resistant subjects (associated with low mortality rates of 40%). It has been reported that genetic polymorphisms conferring resistance to CyHV-3 exist in carp populations (Dixon et al., 2009; Rakus et al., 2009). Of note, epidemiological data suggested that the Eurasian genetic lineages of carp, from which most invasive carp in Australia have descended (Haynes et al., 2012), were largely unaffected by a previous CyHV-3 natural outbreak in Japan (Uchii et al., 2014). Furthermore, wild Australian carp can breed with goldfish (*Carassius auratus*) and produce viable offspring which are expected to have a high resistance to CyHV-3 disease (Kopf et al., 2019) based on studies from the northern hemisphere (Hedrick et al., 2006; Bergmann et al., 2010). Hybridization may introduce resistance alleles into carp populations. If CyHV-3 is released and acts as a selection pressure, it will inevitably induce selection for resistant individuals that will rapidly repopulate the ecosystem. Indeed, carp have an extremely high fecundity and a short generation time. Of note, a mature female carp lays around 100,000–200,000 eggs per kilogram of body weight (Swee and McCrimmon, 1966).

### Effect of Environment on CyHV-3 Disease

Carp is an ectotherm, and as such its biology is highly influenced by the temperature of the water. This environmental factor has been shown to determine the outcome of CyHV-3 infection (Gilad et al., 2003) and has important consequences for biocontrol efficacy (Marshall et al., 2018; Kopf et al., 2019). CyHV-3 can cause disease (i.e., clinical signs and mortalities) at temperatures from 18 to 28°C (Rakus et al., 2013). At low temperatures (< 15°C), carp are sensitive to CyHV-3 infection but they do not express clinical signs under such conditions (Sunarto et al., 2014). Also, the adaptive immune system of carp is down regulated below 14°C (Bly and Clem, 1992), thus individuals infected at this temperature do not acquire a memory immune response and frequently develop disease when returned to permissive temperatures (Sunarto et al., 2014). Above 30°C the virus ceases to replicate and is innocuous to carp. This property has been used in aquaculture to immunize carp against the virus (Ronen et al., 2003). Healthy fingerlings are exposed to the virus by cohabitation with sick fish for 3–5 days at permissive temperature (22–23°C). After that, the fish are transferred to a non-permissive water temperature (≈30°C) for 25–30 days, leading to arrest of viral replication and immunization of the fish.

Ectotherms lack intrinsic thermogenesis and, in most cases, control their body temperature by thermoregulatory movement behavior (Rakus et al., 2017b). In a temperature gradient, ectotherms select a species-specific range of preferred temperature which is defined as “final thermal preferendum.” In response to infection or injection of exogenous pyrogens, ectotherms can increase their body temperature above this final thermal preferendum. This process occurs through behavioral regulation, which leads the animals to move to a warmer environment. This phenomenon is known as “behavioral fever” and is defined as an acute increase of the final thermal preferendum driven by pathogen infection (Evans et al., 2015; Rakus et al., 2017b). Recently, it has been demonstrated that carp infected by CyHV-3 express behavioral fever (Rakus et al., 2017a) by actively seeking warm water. The expression of behavioral fever was shown in laboratory conditions to be beneficial for infected individuals. They all controlled the infection rapidly after expressing behavioral fever, whereas fish maintained at permissive temperature died from the infection (Rakus et al., 2017a).

In combination, the data described above demonstrates the importance of temperature in the outcome of CyHV-3 disease. Whereas aquaculture activities are associated with a rather homogeneous temperature, carp in the wild are exposed to a broad range of temperatures, which could lead to immunization against CyHV-3 rather than to death. An analysis of the temperature variation in the Australian freshwater environment (Becker et al., 2018; Kopf et al., 2019) suggests that this key environmental parameter is likely to negatively affect the impact of CyHV-3 as a biocontrol agent.

### Lessons to be Learned From the Effect of CyHV-3 on Wild Carp Population

The assumption that CyHV-3 could act as an efficient biocontrol agent against wild carp is based on the damage this virus has been causing to the aquaculture sector. However, in natural aquatic ecosystems, carp can move freely, have higher genetic diversity, patchier and lower density, and the environment is more variable and heterogeneous (e.g., water temperature) than in aquaculture ponds or under laboratory conditions (Kopf et al., 2019). Therefore, we expect the mortality of carp, following implementation of the proposed CyHV-3 biocontrol application in the wild, to be much lower and more variable than in laboratory trials and aquaculture ponds (Becker et al., 2018). In support of our hypothesis, it is worth noting that of the few confirmed CyHV-3 carp fish kills in rivers or lakes worldwide, most have had little or no long-term detectable effect on carp abundance (Thresher et al., 2018). For example, Japanese rivers have been monitored for CyHV-3 since 2004, when the virus was associated with mass carp death in Lake Biwa. Ever since, infection has remained widespread without obvious disease outbreaks (Uchii et al., 2014). Finally, several studies have monitored the prevalence of CyHV-3 in Lake Biwa, illustrating a dichotomous nature of viral spread. While young sexually immature carp (< 35 cm) are rarely CyHV-3 infected or seropositive, sexually mature carp (>35 cm) have a very high infection rate (Uchii et al., 2009). This suggests that the breeding behavior of carp promotes CyHV-3 transmission without mortality (Uchii et al., 2011). This phenomenon could be explained, in the wild, by exposure of carp to CyHV-3 during the adult age, i.e., when carp are less sensitive to the disease. Indeed, a strong age-dependent susceptibly of carp to CyHV-3 has been documented (Perelberg et al., 2003).

The observation that CyHV-3 can persist in the wild without inducing carp death suggests that the expression of the disease requires environmental co-factors. Notably, the lack of recorded CyHV-3-associated mass carp deaths in Australia may simply reflect a lack of environmental co-factors rather than the absence of the virus. A recent phylogenetic study suggested that ancestors of CyHV-3 may have infected carp populations, long before the initial outbreaks of the disease were first reported (Gao et al., 2018) and thus long before the introduction of carp in Australia (Kopf et al., 2019). This hypothesis suggests that CyHV-3 could already be present in Australia (Marshall et al., 2018) and that the environmental co-factors found there do not favor the expression of the disease.

## Conclusion

Invasive carp expanded in Australia to their current range and relative abundance decades ago (Shearer and Mulley, 1978; Koehn, 2004). Despite political pressure, there is no environmental justification to rush the release of a viral biocontrol agent. Before continental-scale release of CyHV-3, which would be costly and irreversible, further assessments should include obtaining convincing evidence that prolonged exposure to the virus does not pose a significant risk to non-target native species especially in terms of potential selection for mutations favoring adaptation to such species. While field trials are practicable in endemic areas, they are irrational in a country thought to be free of CyHV-3 and using conditions that cannot guarantee the confinement of the virus. Despite assertions by McColl et al. (2018) that carp biocontrol “is more than just a herpesvirus,” the National Carp Control Plan has no viable plan or technology in place to prevent the immediate recovery of carp populations following potential mortality events. Indeed, an integrated carp management plan should be proposed and implemented. The Daughterless Carp Project is theoretically feasible, but there is presently no large-scale program implementing the production of broodstock or young that would be required for the release. In addition, the long-term safety and efficacy of this technology require further investigation. The authors of this opinion letter represent expertise in intermittent river ecology, aquatic ecology, epidemiology, viral pathogenesis, immunology, herpesviruses, and CyHV-3. We all support the idea that the implementation of a biocontrol measure must be based on robust scientific evidence considering the potential long-term negative effects on the native biodiversity. We do not share the opinion of McColl et al. (2016) who claim “Whatever the nature of the relationship between transmissibility and virulence, the release of CyHV-3 undoubtedly represents a unique and exciting natural experiment that will provide the first opportunity to track, in real time, the co-evolution of both the biocontrol virus and the targeted host.”

## Author Contributions

All authors listed have made a substantial, direct and intellectual contribution to the work, and approved it for publication.

### Conflict of Interest Statement

The authors declare that the research was conducted in the absence of any commercial or financial relationships that could be construed as a potential conflict of interest.
